# Health effects of utilising hospital contacts to provide measles vaccination to children 9–59 months—a randomised controlled trial in Guinea-Bissau

**DOI:** 10.1186/s13063-022-06291-z

**Published:** 2022-04-23

**Authors:** Ane B. Fisker, Justiniano S. D. Martins, Andreas M. Jensen, Cesario Martins, Peter Aaby, Sanne M. Thysen

**Affiliations:** 1 Bandim Health Project, Indepth Network, 1004 Bissau, Guinea-Bissau; 2 Bandim Health Project, University of Southern Denmark, OPEN, 5000 Odense, Denmark; 3 Center for Clinical Research and Prevention, Frederiksberg Hospital, 2000 Frederiksberg, Denmark

**Keywords:** Measles vaccine, Hospital admission, Mortality, Non-specific (heterologous) effects of vaccines

## Abstract

**Background:**

Measles vaccination coverage in Guinea-Bissau is low; fewer than 80% of children are currently measles vaccinated before 12 months of age. The low coverage hampers control of measles. Furthermore, accumulating evidence indicates that measles vaccine has beneficial non-specific effects, strengthening the resistance towards other infections. Thus, even if children are not exposed to measles virus, measles-unvaccinated children may be worse off. To increase vaccination coverage, WHO recommends that contacts with the health system for mild illness are utilised to vaccinate. Currently, in Guinea-Bissau, curative health system contacts are not utilised.

**Methods:**

Bandim Health Project registers out-patient consultations and admissions at the paediatric ward of the National Hospital in Guinea-Bissau. Measles-unvaccinated children aged 9–59 months consulting for milder illness or being discharged from the paediatric ward will be invited to participate in a randomised trial. Among 5400 children, randomised 1:1 to receive standard measles vaccine or a saline placebo, we will test the hypothesis that providing a measles vaccine at discharge lowers the risk of admission/mortality (composite outcome) during the subsequent 6 months by 25%. All enrolled children are followed through the Bandim Health Project registration system and through telephone follow-up. The first 1000 enrolled children are furthermore followed through interviews on days 2, 4, 7 and 14 after enrolment.

**Discussion:**

Utilising missed vaccination opportunities can increase vaccination coverage and may improve child health. However, without further evidence for the safety and potential benefits of measles vaccination, these curative contacts are unlikely to be used for vaccination in Guinea-Bissau.

**Trial registration:**

www.ClinicalTrials.gov NCT04220671. Registered on 5 January 2020.

**Supplementary Information:**

The online version contains supplementary material available at 10.1186/s13063-022-06291-z.

## Introduction

In areas with circulating measles infection, the first dose of measles vaccine (MV) is recommended at 9 months of age and a second dose is recommended later in childhood [1]. To achieve measles control and eventually elimination, countries should achieve at least 95% coverage with both doses [1]. However, Guinea-Bissau and many other countries fall short of this target. Missed opportunities for vaccination are part of the explanation: A recent study from 46 countries showed that 24% of children in contact with the healthcare system during the second year of life were not given one or more missing vaccine doses [2].

**Fig. 1:**
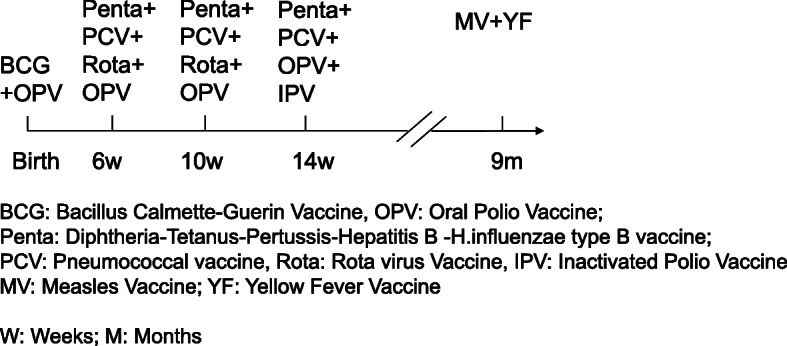
Vaccination schedule in Guinea-Bissau

Though only one dose of MV is scheduled in infancy, the coverage for MV in Africa is lower than the coverage for the third dose of pentavalent (diphtheria, tetanus, pertussis, h. influenzae type B and hepatitis B) vaccine [3] (Fig. 1).

Providing missing doses of MV at health-care contacts has been recommended by WHO for decades [4], but is not universally implemented [5]. At the paediatric ward of the National Hospital Simão Mendes (HNSM) in Guinea-Bissau, no vaccines are currently provided. A reason for not implementing the policy is that health staff and mothers are reluctant to vaccinate ill children. However, a study of adverse events indicates that these were not more common in ill children [6]. In three Southern African studies from the 1960–1980s when measles infection was still a major threat to child mortality, providing MV to children at or during admission to paediatric wards was associated with beneficial effects [7–9]. In the studies with available information on mortality, child mortality was reduced by more than 50% when MV was provided [8, 9].

MV to healthy children in community studies has also been associated with large reductions in child mortality [10–12], likely caused by MV having beneficial non-specific effects (NSE): i.e., the vaccine protects against mortality from other causes than measles [13]. In a recent meta-analysis of observational studies, measles vaccination was associated with 49% (37–58%) lower mortality [14], a reduction which is much larger than could be expected merely from the prevention of measles. We have studied the effect of MV in randomised controlled trials (RCTs) creating extra vaccination opportunities to test the effect of an early additional MV [15–18]. In most of these trials, receiving MV had a marked impact on mortality and very little of the mortality effect was explained by preventing measles infection [15–17]. However, the benefit of MV appears to be sensitive to changes in the sequence of vaccinations: beneficial effects may disappear when non-live vaccines are administered after MV [17, 19] and when children are exposed to campaigns with live oral polio vaccine (OPV) [18, 20]. Hence, to assess the effect of the additional MV, we may have to censor for routine vaccinations and campaigns in the analysis if we have not been able to control their administration in the study design. WHO’s committee on vaccines has recommended more research of the NSE of vaccines [21].

Hence, available data suggest that measles vaccination, also of ill children, may provide a benefit.

## Objectives

To assess the overall health effect of providing measles vaccines at a hospital contact (discharge or outpatient consultation) and thereby obtain evidence for or against implementing the practice.

In a placebo-controlled trial of providing MV to children at hospital contact, we will test the hypothesis that measles vaccination at a curative contact reduces hospital admissions and/or death (composite outcome) by 25% during the subsequent 6 months.

## Methods

### Study setting

The Bandim Health Project (BHP) was initiated in 1978 and now maintains a Health and Demographic Surveillance System (HDSS) in six suburban districts in Bissau, the capital of Guinea-Bissau. The HDSS covers a population of approximately 100,000 individuals and follows children below the age of 3 years through routine home visits with the registration of admission and mortality. The present immunisation schedule in Guinea-Bissau is shown in Fig. 1. The infant immunisation schedule ends with a MV at 9 months of age. However, many children do not receive MV.

BHP registers consultations and hospital admissions of children at HNSM, situated in the centre of Bissau a few kilometres outside the BHP study area. The ward has approximately 100 beds and around 6000 admissions per year for children below the age of 15 [22]. Twenty to 25% of children admitted at HNSM are from the BHP study area [23]. All children coming for consultations or admissions at the hospital pass through the triage room. Subsequently, they are seen by the clinical staff on duty and, if necessary, admitted to the ward. A BHP assistant registers children at the time of triage.

In the triage room, for children aged less than 5 years of age, mothers are asked to present vaccination cards and the vaccination information is copied to the BHP registers. On a daily basis, a team of BHP assistants follow up on all admitted children. Children who did not have their vaccination cards inspected at the date of admission are asked to present their cards during admission.

### Trial design

We will randomise children 1:1 to MV or placebo (saline) at the end of their hospital contact. For hospitalised children, we will enrol and randomise at discharge. For children who have attended outpatient consultations, we will enrol and randomise when they leave the consultation.

### Participants

Measles-unvaccinated children aged 9–59 months are eligible to enter the trial, if they do not have high fever (an axil temperature ≥ 38.0 °C) or a mid-upper-arm-circumference (MUAC) < 110 mm. A MUAC < 110 mm is used to identify children with a high risk of immunodeficiency. Children who did not meet the enrolment criteria at a previous contact can enter the trial at a later contact if they fulfil the enrolment criteria.

### Identification of children to be enrolled and consent process

Following discharge/consultation, mothers/guardians of potentially eligible children will be invited to have their child participate in the trial. A BHP nurse (study staff 1) will provide study information to the mother/guardian orally in Creole and written in Portuguese (official language). It will be explained that MV is recommended to all measles-unvaccinated children at contacts with the health system, but that it is currently not implemented in Guinea-Bissau. To test whether a MV can reduce the risk of becoming ill again, we will vaccinate children with MV or placebo. It will be emphasised that the injection given on the day of enrolment does not replace the routine MV that children should otherwise receive, and mothers/guardians will be told that these vaccines can be obtained at the health centres. To avoid giving two vaccines close to another we will recommend that mothers wait 2 weeks before seeking measles vaccination. Furthermore, it is emphasised that information collected by the BHP staff will be treated confidentially. The information sheet with trial information will be fixed to the child’s vaccination card.

If the mother/guardian wishes her child to participate in the trial, informed consent will be documented by signature or fingerprint. The signature of an independent person will certify the fingerprint. Provided the mother/guardian consents to have her child participating in the trial, the enrolling nurse (study staff 1) measures and documents MUAC and temperature and verifies that the child fulfils the enrolment criteria. For all enrolled children a sticker with a unique study number will be affixed to the child’s vaccination card (or a replacement card for children who have lost their vaccination card).

### Enrolment

In addition to MUAC and temperature, the enrolling nurse (study staff 1) collects information on prior admissions, symptoms at the time of enrolment, prescribed treatment and takes a photo of the area of the vaccination card, where the vaccines are documented. Through the routine monitoring system at HNSM, information on vaccination status, whether the child has a scar after Bacillus Calmette-Guérin vaccination, weight, maternal age, ethnicity, and education is collected.

The mother/guardian then picks a numbered envelope from a randomisation bag. The number of the envelope is noted in the enrolment form. The sealed envelope is then passed on to another staff member responsible for preparing the vaccine/placebo (study staff 2, see below).

### Implementation of randomisation and blinding of participants

Randomisation lists are prepared using computer-generated random numbers. The programme allocates a list of 12 randomisation numbers to two different groups. Separate blocks are prepared for boys and girls. The randomisation key is stored in a table, which is deposited with the Data Safety and Monitoring Board (DSMB).

Randomisation lots (three adhesive labels: two labels both numbered with the block number and one of the numbers 1–12; one of the labels additionally carrying the group allocation) are printed and packed in envelopes by staff not involved in the randomisation process. The block and sequence number are written on the envelope and the envelope is sealed. Twelve randomisation envelopes from the same block (6 MV, 6 placebo) are placed in a bag.

Randomisation is implemented by the mother/guardian picking an envelope from a randomisation bag according to the sex of the child. A new bag is not opened until the prior bag is empty. Thus, we ensure that randomisation is balanced over time and by sex.

To maintain blinding, we involve two members of the study staff in the randomisation and vaccination procedure: At enrolment, the mother/guardian picks an envelope from the randomisation bag, and study staff 1 transfers the number from the outside of the envelope to the consent form and enrolment record. The envelope is then given to study staff 2.

In another room, study staff 2 opens the envelope and prepares a syringe with MV or saline dependent on the information in the envelope. The label with group allocation is put onto the study log with information on the vial number of MV or placebo, batch number, expiration date and the information on the time of preparing the vaccine. The other label with the envelope number is affixed to the syringe, placed inside the envelope in a cooling box and returned to study staff 1.

On reception of a numbered syringe, the enrolling nurse verifies the envelope number and administers the injection (0.5 ml saline or MV) as a subcutaneous injection in the scapular region.

### Follow up and assessment of outcomes

Enrolled children will be followed through passive case detection as all admissions to the paediatric ward of HNSM are registered by the BHP. Telephone interviews will be conducted for all children at 3, 6 and 12 months after enrolment to enquire about admissions and mortality (Table 1). For all registered deaths, a home visit to conduct a structured interview (a verbal autopsy [24]) will be performed. Through this interview, parents/caretakers of a deceased child are asked to provide information on the circumstances and symptoms leading up to the death. Based on the verbal autopsy, we will classify the cause of death and validate the information obtained through the telephone interview.

**Table 1:**
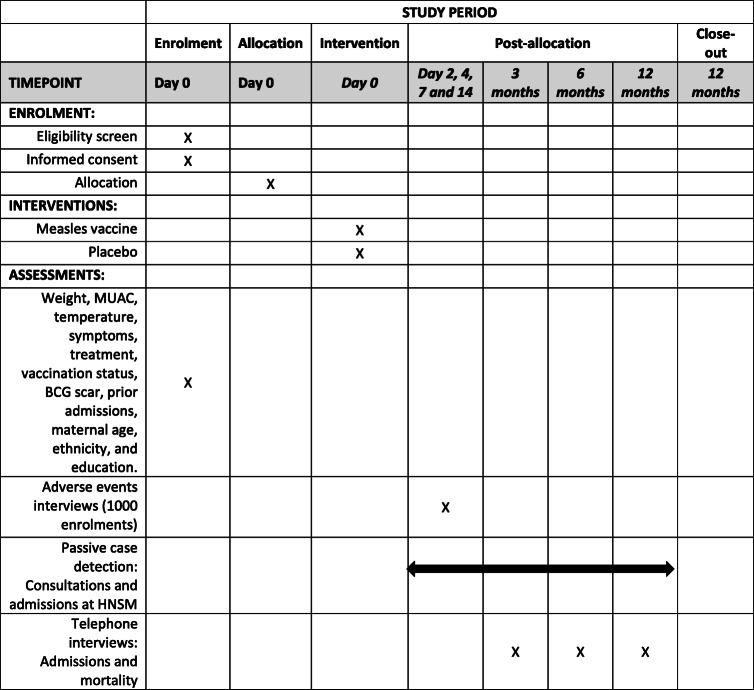
Timeline for recruitment, assessments, and interventions

Note: *MUAC* mid upper-arm circumference, *BCG* bacillus Calmette-Guérin vaccine, *HNSM* National Hospital Simão Mendes

The studies that tested measles vaccination in ill children have found that vaccination of ill children does not pose a risk to the child [7–9] and that the mild adverse events are no more common in ill children [6]. Severe adverse events (admissions and mortality) are the main outcomes of the trial and will be assessed through the trial.

Children from the BHP study area will furthermore be followed through the BHP routine registration system, which allows both validating the information obtained through the telephone interviews with information obtained through the BHP HDSS, assessing potential mild adverse reactions leading to the health system contacts and registering subsequent vaccines.

To ensure the safety of children in our trial, we will additionally contact the first 1000 enrolled children 2, 4, 7 and 14 days after enrolment, and hence also collect information before the telephone call 6 months after enrolment. Furthermore, during the first months, we will only enrol children living in or close to the BHP study area and follow them through home visits. Subsequently, children living outside the study area will also be enrolled. They will be followed through phone calls.

### Primary outcome

Our primary outcome is a composite outcome of non-accidental death or an identified non-accidental hospital admission at HNSM within 6 months after enrolment. We have defined the primary outcome as a composite outcome of mortality and admissions as many deaths occur at home and would therefore not be identified through HNSM data. Since children are enrolled at the national hospital, we expect that guardians of children experiencing severe illness will also seek health care at HNSM for subsequent episodes. For the primary outcome, we will censor follow-up at 6 months. We anticipate a stronger effect during the first months before subsequent vaccines may dilute an effect. Follow-up will continue for 12 months and be reported in a secondary analysis. Since we rely on passive case detection at HNSM, we will not censor the analysis time of children with no information by telephone interviews before 6 (﻿12) months in the primary (secondary) analysis.

### Secondary outcomes

The secondary outcomes are:

- Non-accidental mortality within 6 and 12 months after enrolment. Censoring will be applied for children for whom we do not obtain information by telephone.
- Non-accidental hospital admission with an overnight stay in any health facility within 6 months after enrolment, excluding the first 2 weeks after enrolment. Censoring will be applied for children for whom we do not obtain information by telephone.
- Cause-specific mortality and/or hospital admissions at the HNSM (classifying admissions in the main categories: respiratory infections, Gastro-intestinal infections, sepsis, malaria and others)
- Adverse events were assessed through registered contacts with the health system (information on consultations or admissions during the first 2 weeks after enrolment identified through the HNSM registration system and registration of outpatient consultations at the health centres in the study area). If the health of a child does not improve after an out-patient consultation, the child will often be brought for a new consultation. We will therefore specifically examine whether randomisation status affects the risk of re-consultations.
- Cost-effectiveness of providing MV at hospital contacts analysed using a societal perspective (provided we find evidence of a beneficial effect). By using the societal perspective, we will take into account both direct costs/savings experienced by the individual and the health care system, and the social opportunity costs such as lost productive time of the mother due to caring for an ill child [25].

### Sample size

We have previously observed that 8% of children admitted between 9 and 18 months experience a subsequent admission before 18 months of age [26]. We therefore expect that the rate of events in the present trial is at least 8% during the subsequent 6 months among measles-unvaccinated children. A prior trial found 30% (5–45%) lower risk of admission after a first MV [27]. Effects on mortality have been similar or stronger [15, 16], but were not confirmed in recent trials [18, 28], potentially due to interactions with frequent campaigns with OPV [20]. In the present trial where we censor on vaccination campaigns after enrolment, we will have 80% power to detect a 25% difference in admission rates, if we enrol 2653 children in each study arm.

We currently inspect vaccination cards for 2/3 of all children aged 9–59 months, but we assume that this proportion can be increased. Since all routine vaccines in Guinea-Bissau are administered at distinct anatomic locations (MV being given as a subcutaneous injection in the subscapular region), we will use maternal information if the vaccination card of the child is stated to have been lost. A prior study has indicated accurate recall of measles vaccination status [29].

Based on data from the paediatric ward, we should be able to enrol 165 measles-unvaccinated children, who have presented a vaccination card, per month. By including also children who presented no vaccination card but were reported to be measles-unvaccinated, we expect that we will need 24 months to enrol a total of 5400 children. If the first year of enrolment and follow-up indicates lower outcome rates, possible resizing of the trial will be discussed with the DSMB.

### Data management

Trial data collected at enrolment, during follow-up home visits and during phone calls are entered in customised forms build in ODK-X with build-in range and condition checks. Data collection is performed by trained BHP field workers and takes place using password-protected Android tablets which are kept under lock when not in use. During data collection, data are stored in a local SQLite database on the tablet. Data are synchronised with the central database at the end of every day’s work. The data transfer process is secured with SSL. The central database is password protected. Stored data will only be pseudonymised at the time of data lock to enable linkage to the HDSS data and the HNSM databases. After pseudonymisation, the locked datasets will be linked to the randomisation table for analysis.

### Statistical methods

We will use Cox proportional hazards models to estimate hazard ratios with age as the underlying timescale to compare event rates between children in the intervention and control groups. In the primary analysis, children will be followed from enrolment to first non-accident admission or death within 6 months after enrolment. Should national vaccination campaigns targeting the enrolled children be implemented during the course of this trial, follow-up for the main analysis will be censored at the beginning of the campaign (on an intention-to-treat basis). Detailed statistical methods for both primary and secondary outcomes are described in the analysis plan (Additional file 1).

Prior studies have indicated that MV is particularly beneficial for girls [16, 30]. We will investigate whether effects differ by sex. MV may have a stronger effect on admissions in the dry season [27]. We will investigate interactions with both season of enrolment and season of time at risk. Furthermore, receiving delayed pentavalent vaccine after MV has been associated with increased risk of mortality and admission [19, 31]. Exposure to OPV campaigns before measles vaccination may reduce the benefit of MV [20]. We will therefore assess interaction with pre-enrolment vaccination status.

The effects of the intervention on mortality, admissions and cause-specific composite outcome will be analysed in similar Cox proportional hazards models. The proportion of children having sought consultations during the first two weeks will be compared in binomial regression models (Additional file 1).

Sensitivity analyses: Children who have been eligible for MV in a campaign (but have received no routine MV) will be eligible to enter the trial but will be excluded in a sensitivity analysis. We will explore whether an effect changes over time by splitting the analysis time after the first 3 months. Furthermore, we will assess whether changing the underlying timescale to time since enrolment alters conclusions.

### Monitoring

A DSMB consisting of a paediatrician (Poul-Erik Kofoed, professor of paediatrics, Sygehus Lillebælt, Denmark), a statistician (Tuomo Nieminen, THL, Finland) and an epidemiologist (Katrine Hass Rubin, associate professor, OPEN, University of Southern Denmark) has been formed. The DSMB advises the investigators and will receive a written report of the current status of the trial every 6 months. Any changes to the protocol will be discussed with the DSMB prior to submitting an amendment request to the Guinean Ethics Committee. Following approval, changes will be made in the trial registry at www.clinicaltrials.gov.

### Safety and Interim analyses

The DSMB will hold the randomisation code (randomisation lot numbers and group allocation). Based on tables with enrolments and registered events provided to the statistician of the DSMB after the enrolment of 1000 children, 50% and 75% of children, the DSMB-statistician performs an interim analysis at these three timepoints during the trial. The table with registered events will be compiled based on data from the surveillance from the paediatric ward at HNSM, the telephone follow-up, the HDSS monitoring and the adverse events follow-up.

If the interim analysis indicates a strong difference (*p* < 0.001), the DSMB asks the study team to stop enrolment.

As a public financed Danish research institution, the University of Denmark is self-insured and cannot take out a liability insurance through a private company. As an investigator-initiated trial by investigators affiliated with the University of Southern Denmark, any harm to study participants due to their participation in the trial is thus covered by the University of Southern Denmark.

## Discussion

In the present trial, we aim to study the impact of an already recommended policy of providing measles vaccine at contacts with the health care system. It can be discussed if it is needed to conduct such a trial of an already recommended policy. We do think so, as this policy is not current practice in many countries. Unless we can provide solid evidence that there are substantial benefits connected with utilising these contacts, we are unlikely to alter the implementation of policy.

We opted to use a saline placebo to increase the impact of the study. Using an alternative vaccine (instead of saline) could potentially hamper conclusions, as another vaccine may also have NSEs and hence affect health outcomes (positively or negatively) in the placebo arm [32].

Due to blinding, a child who is measles vaccinated at enrolment may subsequently be brought for vaccination. WHO recommends measles vaccination of children during campaigns regardless of the interval to the prior dose, and a short interval is not considered to pose a risk to the child [33]. However, due to concerns voiced by the National Ethics Committee in Guinea-Bissau, we will recommend mothers to wait 2 weeks before bringing their children for vaccination. We have planned intensive follow up of the first 1000 enrolled children to ensure that we detect and can act on adverse events.

This trial will provide a conservative estimate of the effect of providing MV at hospital contacts, as some children in the control group may be vaccinated shortly after enrolment. It will assess the overall health effect of a current policy that is not implemented in many countries. This trial may provide countries with an incentive to reduce missed vaccination opportunities and exploit hospital contacts to provide vaccination. If the effect is around the same magnitude as we have observed in prior studies, the impact on child mortality will be substantial.

The intervention is an easy-implementable add-on to the current practices in many countries. At the same time, it will strengthen our knowledge about the NSEs of MV. For the last many years there have been no measles circulating in Bissau, hence any health benefit observed is likely to be due to NSEs.

Furthermore, we will provide cost-effectiveness estimates of whether it is cost-effective to provide measles vaccination at hospital contacts. In a prior cost-effectiveness study from Guinea-Bissau, we showed that it is cost-effective to open a vial of MV for every child even when more than 8 of 10 doses are wasted [34]. In the planned trial, the additional cost of measles vaccination is anticipated to be even lower, and thus the trial may provide further support for changes in policy implementation.

## Dissemination plans

The findings of this randomised trial will be published in international peer-reviewed journals. Authorship criteria will follow ICMJE guidelines. Results with direct implications for WHO vaccination policy will be communicated directly to WHOs Strategic Advisory Board of Experts on Immunization. We have a close collaboration with the national health authorities in Guinea-Bissau, and the results will be shared with the relevant institutions.

## Trial status

Enrolment was initiated in January 2020 but paused in March 2020 due to the corona pandemic. We anticipate to complete enrolment by December 2023.

## Supplementary Information

**Additional file 1.** Appendix. Analysis plan.

**Additional file 2.**

## Data Availability

Data will be available on a collaborative basis through contact to the Bandim Health Project.

## Abbreviations

BHP: Bandim Health Project
DSMB: Data Safety and Monitoring Board
HDSS: Health and Demographic Surveillance System
HNSM: Hospital National Simão Mendes (the national hospital in Guinea-Bissau)
MUAC: Mid-upper-arm-circumference
MV: Measles vaccine
NSE: Non-specific effects
OPV: Oral polio vaccine
RCT: Randomised controlled trial
WHO: World Health Organization
