# Supplementary material for: Health effects of utilising hospital contacts to provide measles vaccination to children 9–59 months—a randomised controlled trial in Guinea-Bissau
Source: Trials. 2022 Apr 23;23:349. doi: 10.1186/s13063-022-06291-z (PMC9034539; doi:10.1186/s13063-022-06291-z)
Supplement: Supplementary file 1 — Additional file 1. Appendix. Analysis plan. [file 13063_2022_6291_MOESM1_ESM.docx]

**Analysis plan****:**

**Health Effects of Utilising Hospital Contacts to Provide Measles Vaccination to children 9-59 months – a Randomised Controlled Trial in Guinea-Bissau**

Version 28-02-2022

Contents

[1. General analysis principles 2](#_Toc96958806)

[1.1 Participant population 2](#_Toc96958807)

[1.2 Multiple testing 2](#_Toc96958808)

[1.3 Missing data 2](#_Toc96958809)

[1.4 Test for proportional hazards 2](#_Toc96958810)

[2. Analyses 3](#_Toc96958811)

[2.1 Baseline comparison 3](#_Toc96958812)

[2.2 Primary analysis of primary outcome 4](#_Toc96958813)

[2.3 Effect-modifier analyses of primary outcome. 5](#_Toc96958814)

[**2.3.1. Background factors known at enrolment** 5](#_Toc96958815)

[**2.3.2. Changing effects during follow up – exposure to campaigns** 6](#_Toc96958816)

[**2.3.3. Changing effects during follow up – Season of follow-up** 7](#_Toc96958817)

[2.4. Secondary outcomes 8](#_Toc96958818)

[**2.4.1. Non-accidental mortality within 6 and 12 months of follow-up.** 8](#_Toc96958819)

[**2.4.2. Non-accidental admissions (any health facility) within 6 months of follow-up.** 8](#_Toc96958820)

[**2.4.3. Cause specific mortality or admissions at HNSM within 6 months of follow-up.** 9](#_Toc96958821)

[**2.4.4. Short term adverse events leading to contact with the health system** 9](#_Toc96958822)

[2.5 Bayesian analysis of primary outcome 10](#_Toc96958823)

[2.6 Sensitivity analyses 10](#_Toc96958824)

[2.7 Cost-effectiveness of providing MV at curative health contacts 11](#_Toc96958825)

# General analysis principles

## 1.1 Participant population

All analyses will be conducted on the per-protocol population.

## 1.2 Multiple testing

P-values will not be corrected for multiple testing. Secondary outcomes are tested to examine if the pattern is similar across other measures of child health. Consequently, p<=0.05 will not be employed as a threshold for statistical significance for secondary outcomes. For the sensitivity analyses, we will not consider statistical significance, but rather robustness of the conclusions across different definitions of outcomes and co-variates.

## 1.3 Missing data

All analyses will be complete-case analyses. However, if we have no information on a particular event being caused by accident, we will assume that it was not. Missing information due to not being able to contact participants by telephone does not affect the main outcome measure.

## 1.4 Test for proportional hazards

To test the proportional hazards assumption, a required assumption of the Cox regression, we will perform formal significance tests based on Schoenfeld residuals and graphically via log-log survival curves. In addition, we will assess proportionality by allowing the hazard ratio to interact with the underlying timescale to identify a possible time trend.

Significance tests based on Schoenfeld residuals will be performed via the stata command *estat phtest, detail* leading to both a global test and a test for each covariate, the latter being relevant only when we study effect modifications. Presentation of log-log survival curves will be undertaken via *stphplot.* Finally, possible interactions between hazard ratios and the underlying time scale will be further investigated via the *stcox* procedure and the *tvc()* option. For the models including effect modifications we will construct a new interaction variable (i.e., a four-level variable representing the interaction) such that a graphical assessment of proportionality can be undertaken assessing the four-level variable in a log-log survival plot*.*

If we identify evidence for non-proportionality, we will still report the marginal hazard ratios but supplement this measure by hazard ratios for 2-3 properly selected categorical time-periods identified based on the aforementioned proportionality investigations.

# 2. Analyses

## 2.1 Baseline comparison

Descriptive statistics:

We will describe participant flow by group allocation in a flowchart. For children included in the main analysis, we will describe background factors. Distribution of background factors will be presented by group allocation. Background factors will be summarised by counts (percentages), means (standard deviation) or medians (interquartile range) as appropriate. Information on the proportion with missing information will be provided.

**Table 1: Summary of background factors by intervention and control group**

| - Sex - Age - Enrolled after admission/Outpatient consultation - Mid-upper-arm circumference and weight-for-age - Vaccination status for other routine vaccines (Reception and timeliness) - Maternal factors (age, education, BCG scar) - Prior admissions |
| --- |

## 2.2 Primary analysis of primary outcome

Our primary outcome is a composite outcome of non-accidental death or an identified non-accidental hospital admission at HNSM within 6 months after enrolment. The primary analysis of the primary outcome is described in more detail in Table 2.

**Table 2: Primary analysis of primary outcome**

| Population | Per-protocol population |
| --- | --- |
| Observation period | From: enrolment  To: 183 days after enrolment  Censoring: Date of exposure to national vaccination campaign after enrolment. Death due to accident |
| Failure definition | Death (any location) or first post-enrolment admission at HNSM not classified as caused by accidents. |
| Statistical tool | Cox proportional hazards model |
| Stratification | Sex (as randomisation stratified by sex) |
| Outline stata code  For analysis: | stset outdate, f(combinedoutcome=1) origin(datebirth) enter(dateenrol) exit(dateenrol+183)  stcox group, strata(sex)  Check of proportional hazards assumption^[[1]](#footnote-1)^:  estat phtest, detail  stphplot, strata(group) adj(sex)^[[2]](#footnote-2)^  stcox group, strata(sex) tvc(group) texp(_t) |

## 2.3 Effect-modifier analyses of primary outcome.

### **2.3.1. Background factors known at enrolment**

We will assess whether the effect of the intervention on the primary effect measure is modified by the potential effect modifiers sex, season of enrolment, reception of the third dose of pentavalent vaccine (Penta3) and vaccination campaigns.

**Table 3. Potential effect modifiers of the primary outcome – fixed during observation period**

| Potential effect modifiers | Sex  Season of enrolment (Rainy: June-November, Dry: December-May)  Penta3 prior to enrolment (Received / Not received)  Exposure to vaccination campaigns with oral polio vaccines (OPV) prior to enrolment |
| --- | --- |
| Observation period | From: enrolment  To: 183 days after enrolment  Censoring: Date of exposure to national vaccination campaign after enrolment. Death due to accident |
| Failure definition | Deaths (any location) or admissions at HNSM not classified as caused by accidents. |
| Statistical tool | Cox proportional hazards model |
| Reasoning | Prior studies have indicated that MV is particularly beneficial for girls^1 2^, and we will investigate if effects differ by sex.  MV may have a stronger effect on admissions in the dry season, and we will investigate interactions with both season of enrolment and season of time at risk^3^.  Furthermore, receiving inactivated delayed pentavalent vaccine after MV has been associated with increased risk of mortality and admission^4 5^. Exposure to OPV campaigns prior to enrolment have in a prior trial counteracted a beneficial effect of MV^6^.  We will therefore assess interaction with pre-enrolment vaccination status. |
| Stratification | Sex (as randomisation is stratified by sex) |
| Outline stata code  For analysis: | stset outdate, f(combinedoutcome=1) origin(datebirth) enter(dateenrol) exit(dateenrol+183)  stcox group#EM EM, strata(sex)   - EM=the potential effect modifier (categorical variable)   Check of proportional hazards assumption:  estat phtest, detail  stphplot, strata(group EM) adj(sex)  stcox group#EM EM, strata(sex) tvc(group#EM EM) texp(_t) |

### **2.3.2. Changing effects during follow up – exposure to campaigns**

In the main analyses, observation time will be censored at exposure to vaccination campaign during follow up. However, to investigate if MV modifies the response to subsequent campaigns, we will also investigate interactions.

**Table 4. Potential effect modifiers of the primary outcome – Exposure to campaigns during observation period**

| Potential effect modifiers | Campaigns implemented during follow-up |
| --- | --- |
| Observation period | From: enrolment  To: 183 days after enrolment  Censoring: Death due to accident |
| Failure definition | Deaths (any location) or admissions at HNSM not classified as caused by accidents. |
| Statistical tool | Cox proportional hazards model |
| Reasoning | Prior studies have indicated that subsequent campaigns may modify the effect of MV^6^. |
| Stratification | Sex (as randomisation stratified by sex) |
| Outline stata code  For analysis: | stset outdate, f(combinedoutcome=1) origin(datebirth) enter(dateenrol) exit(dateenrol+183) id(studynumber)  g cdate=date-of-campaign if dateenrol< date-of-campaign & datebirth+_t> date-of-campaign  “date-of-campaign” = first date eligible for participation in a campaign after enrolment  stsplit camp, at(0) after(cdate)  replace camp=camp+1  recode camp .=0  stcox group#camp camp, strata(sex)  Check of proportional hazards assumption:  estat phtest, detail  stphplot, strata(group camp) adj(sex)  stcox group#camp camp, strata(sex) tvc(group#camp camp) texp(_t) |

### **2.3.3. Changing effects during follow up – Season of follow-up**

Mortality levels^7^ and pathogen exposure^8 9^ vary by season. We will investigate if the effect of MV vary by season of exposure.

**Table 5. Potential effect modifiers of the primary outcome – Season of follow-up**

| Potential effect modifiers | Season of follow-up |
| --- | --- |
| Observation period | From: enrolment  To: 183 days after enrolment  Censoring: Date of exposure to national vaccination campaign after enrolment. Death due to accident |
| Failure definition | Deaths (any location) or admissions at HNSM not classified as caused by accidents. |
| Statistical tool | Cox proportional hazards model |
| Reasoning | Prior studies have indicated that subsequent campaigns may modify the effect of MV^6^. |
| Stratification | Sex (as randomisation stratified by sex) |
| Outline stata code  For analysis: | stset outdate, f(combinedoutcome=1) origin(datebirth) enter(dateenrol) exit(dateenrol+183) id(studynumber)  g seasonshift=.  foreach date in d(01/05/2020) d(01/12/2020) d(01/05/2021) d(01/12/2021) d(01/05/2022) d(01/12/2022) {  replace seasonshift=’date´ if dateenrol<’date´ &datebirth+_t>’date´&’date´==.  stsplit sshift, at(0) after(seasonshift)  replace sshift = sshift +1  recode sshift .=0  gen seasonfu=seasonenr+sshift if seasonenr==0  replace seasonfu=seasonenr-sshift if seasonenr==1   - Where seasonenr is season enrolment (0/1)   stcox group#seasonfu seasonfu, strata(sex)  Check of proportional hazards assumption:  estat phtest, detail  stphplot, strata(group seasonfu) adj(sex)  stcox group#seasonfu seasonfu, strata(sex) tvc(group#seasonfu seasonfu) texp(_t) |

## 2.4. Secondary outcomes

### **2.4.1. Non-accidental mortality within 6 and 12 months of follow-up.**

In addition to the composite outcome, we will investigate the effect on the separate components: Mortality and admission.

**Table 6: Secondary outcome: Non-accidental mortality.**

| Population | Per-protocol population |
| --- | --- |
| Observation period | From: enrolment  To: 183 days after enrolment (and 365.25 days after enrolment)  Censoring: Date of exposure to national vaccination campaign after enrolment. Death due to accident |
| Failure definition | Deaths (any location) not classified as caused by accidents. |
| Statistical tool | Cox proportional hazards model |
| Stratification | Sex (as randomisation stratified by sex) |
| Outline stata code  For analysis: | stset outdate, f(death=1) origin(datebirth) enter(dateenrol) exit(dateenrol+183)   - and   stset outdate, f(death=1) origin(datebirth) enter(dateenrol) exit(dateenrol+365.25)  stcox group, strata(sex)  Check of proportional hazards assumption:  estat phtest, detail  stphplot, strata(group) adj(sex)  stcox group, strata(sex) tvc(group) texp(_t) |

### **2.4.2. Non-accidental admissions (any health facility) within 6 months of follow-up.**

In addition to the composite outcome, we will investigate the effect on the separate components. Hospital admissions will be analysed as repeated events, censoring the first 2 weeks after enrolment.

**Table 7: Secondary outcome: Non-accidental admission.**

| Population | Per-protocol population |
| --- | --- |
| Observation period | From: 2 weeks after enrolment  To: 183 days after enrolment  Censoring: Death or last date of obtaining information by phone call. Date of exposure to national vaccination campaign after enrolment. |
| Failure definition | Admission not classified as caused by accidents, with overnight stay in any location. |
| Statistical tool | Cox proportional hazards model |
| Stratification | Sex (as randomisation stratified by sex) |
| Outline stata code  For analysis: | stset outdate2, f(admission=1) origin(datebirth) enter(dateenrol+14) exit(dateenrol+183) time0(indate) exit(censoring_date) id(study number)   - where: outdate2 is date of last interview/registered admission; - indate is date of becoming at risk (i.e., date of enrolment+14 or date of discharge after a previous admission)   stcox group, strata(sex)  Check of proportional hazards assumption:  estat phtest, detail  stphplot, strata(group) adj(sex)  stcox group, strata(sex) tvc(group) texp(_t) |

### **2.4.3. Cause specific mortality or admissions at HNSM within 6 months of follow-up.**

We will investigate whether the effect of MV varies for different causes of mortality/admission

**Table 8: Cause specific outcomes**

| Population | Per-protocol population |
| --- | --- |
| Observation period | From: enrolment  To: 183 days after enrolment  Censoring: Date of exposure to national vaccination campaign after enrolment. Death due to accident. |
| Failure definition | Mortality or admission with overnight stay in HNSM classified as caused by X. (Information obtained through verbal autopsy and hospital records)  Where X= Respiratory infections, Gastro-intestinal infections, Sepsis, Malaria and other |
| Statistical tool | Cox proportional hazards model |
| Stratification | Sex (as randomisation stratified by sex) |
| Outline stata code  For analysis: | stset outdate, f(combinedoutcome=1&cause==X) origin(datebirth) enter(dateenrol) exit(dateenrol+183) id(studynumber)  stcox group, strata(sex)  Check of proportional hazards assumption:  estat phtest, detail  stphplot, strata(group) adj(sex)  stcox group, strata(sex) tvc(group) texp(_t) |

### **2.4.4. Short term adverse events leading to contact with the health system**

To ensure the safety of children in our trial, we will additionally contact the first 1000 enrolled children 48 hours, 4, 7 and 14 days after enrolment. At home visits/telephone interviews information on contacts with the health system is collected.

In the BHP study area and at the HNSM information on all contacts are routinely collected.

**Table 9: Health Facility Consultations.**

| Population | 1. Per-protocol population followed intensively during the initial phase of the trial 2. Per-protocol population resident in the urban BHP study area. 3. Per-protocol population |
| --- | --- |
| Observation period | From: enrolment  To: 14 days after enrolment |
| Failure definition | 1. Reported consultations 2. Registered contacts with the health system identified through the HNSM registration system and registration of outpatient consultations at the health centres in the study area. 3. Registered contacts with the health system identified through the HNSM registration system |
| Statistical tool | Binomial regression |
| Stratification | Sex (as randomisation stratified by sex) |
| Outline stata code  For analysis: | binreg contact group sex, rr |

## 2.5 Bayesian analysis of primary outcome

In addition to the planned frequentist analysis, we plan a Bayesian analysis of our primary outcome to calculate the probability of any reduction of events.

For the analysis described in detail in Table 10, we have chosen weakly informed priors as previous studies have indicated beneficial effects of MV (particularly for girls).

**Table 10: Bayesian survival analysis of primary outcome**

| Population | Per-protocol population |
| --- | --- |
| Observation period | From: enrolment  To: 183 days after enrolment  Censoring: Date of exposure to national vaccination campaign after enrolment. Death due to accident |
| Failure definition | Death (any location) or a post-enrolment admission at HNSM not classified as caused by accidents. |
| Statistical tool | Bayesian survival analysis (Weibull distribution) |
| Stratification | Sex (as randomisation stratified by sex) |
| Outline stata code  For analysis: | stset outdate, f(combinedoutcome=1) origin(datebirth) enter(dateenrol) exit(dateenrol+183)  bayes, normalprior(10): streg combinedoutcome i.group, ///  distribution(weibull) strata(sex) eform  Probability for any effect:  bayestest interval ({_t:combinedoutcome}), upper(0)  Model check:  bayesgraph diagnostic _all |

## 2.6 Sensitivity analyses

We will investigate if the effect varies during the time of follow up, splitting observation time after 3 months, as well as splitting the observation time after 1 month as some adverse events may occur late. Furthermore, we will assess whether changing the underlying timescale to time since enrolment alters conclusions.

Children who have been eligible for MV in a campaign (but have received no routine MV) will be eligible to enter the trial but will be excluded in a sensitivity analysis.

## 2.7 Cost-effectiveness of providing MV at curative health contacts

A cost effectiveness analysis seeking to measure the cost per death averted using a societal perspective will be performed, contrasting the costs of vaccine provision at curative contacts with the current scenario. The costs/savings associated with potential different rates of admissions will also be taken into account.

**References**

1. Aaby P, Martins CL, Garly ML, et al. Non-specific effects of standard measles vaccine at 4.5 and 9 months of age on childhood mortality: randomised controlled trial. *BMJ* 2010;341:c6495. doi: 10.1136/bmj.c6495 [published Online First: 2010/12/02]

2. Fisker AB, Hornshoj L, Rodrigues A, et al. Effects of the introduction of new vaccines in Guinea-Bissau on vaccine coverage, vaccine timeliness, and child survival: an observational study. *The lancet global health* 2014;2(8):e478-87. doi: 10.1016/S2214-109X(14)70274-8 [published Online First: 24-07-2014]

3. Martins CL, Benn CS, Andersen A, et al. A randomized trial of a standard dose of Edmonston-Zagreb measles vaccine given at 4.5 months of age: effect on total hospital admissions. *J Infect Dis* 2014;209(11):1731-8. doi: 10.1093/infdis/jit804

4. Fisker AB, Thysen SM. Non-live pentavalent vaccines after live measles vaccine may increase mortality. *Vaccine* 2018;36(41):6039-42. doi: 10.1016/j.vaccine.2018.08.083 [published Online First: 2018/09/10]

5. Biai S, Rodrigues A, Nielsen J, et al. Vaccination status and sequence of vaccinations as risk factors for hospitalisation among outpatients in a high mortality country. *Vaccine* 2011;29(20):3662-9. doi: 10.1016/j.vaccine.2011.03.016 [published Online First: 2011/03/29]

6. Aaby P, Andersen A, Martins CL, et al. Does oral polio vaccine have non-specific effects on all-cause mortality? Natural experiments within a randomised controlled trial of early measles vaccine. *BMJ open* 2016;6(12):e013335. doi: 10.1136/bmjopen-2016-013335

7. Nielsen BU, Byberg S, Aaby P, et al. Seasonal variation in child mortality in rural Guinea-Bissau. *Trop Med Int Health* 2017;22(7):846-56. doi: 10.1111/tmi.12889

8. Rodrigues A, de Carvalho M, Monteiro S, et al. Hospital surveillance of rotavirus infection and nosocomial transmission of rotavirus disease among children in Guinea-Bissau. *Pediatr Infect Dis J* 2007;26(3):233-7. [published Online First: 2007/05/09]

9. Rodrigues A, Schellenberg JA, Kofoed PE, et al. Changing pattern of malaria in Bissau, Guinea Bissau. *TropMedIntHealth* 2008;13(3):410-17.

1. If we identify evidence for non-proportionality, we will still report the marginal hazard ratios, but supplement this estimate by hazard ratios for 2-3 properly selected categorical time-periods identified based on the aforementioned proportionality investigations [↑](#footnote-ref-1)
2. If there is indication of non-proportionality based on assessment of the log-log curves, we will test for non-proportionality by replacing the term texp(_t) with texp(_t>s) for specific values of s. [↑](#footnote-ref-2)
